# Propionate and Alzheimer’s Disease

**DOI:** 10.3389/fnagi.2020.580001

**Published:** 2021-01-11

**Authors:** Jessica Killingsworth, Darrell Sawmiller, R. Douglas Shytle

**Affiliations:** Department of Neurosurgery and Brain Repair, Morsani College of Medicine, University of South Florida, Tampa, FL, United States

**Keywords:** gut microbiome, valproate, short chain fatty acids, Alzheimer’s disease, propionate

## Abstract

Propionate, a short-chain fatty acid, serves important roles in the human body. However, our review of the current literature suggests that under certain conditions, excess levels of propionate may play a role in Alzheimer’s disease (AD). The cause of the excessive levels of propionate may be related to the *Bacteroidetes* phylum, which are the primary producers of propionate in the human gut. Studies have shown that the relative abundance of the *Bacteroidetes* phylum is significantly increased in older adults. Other studies have shown that levels of the *Bacteroidetes* phylum are increased in persons with AD. Studies on the diet, medication use, and propionate metabolism offer additional potential causes. There are many different mechanisms by which excess levels of propionate may lead to AD, such as hyperammonemia. These mechanisms offer potential points for intervention.

## Introduction

Dietary fibers are carbohydrate polymers that have at least 10 monomeric units (Cummings et al., 2009). The enzymes needed to digest most dietary fibers are lacking in the human body (den Besten et al., 2013). Therefore, the microbiota in the intestine is tasked with fermenting dietary fibers. Fermentation results in the production of short-chain fatty acids (SCFAs), which serve several important functions. In the gut, they aid in microbial growth (Alexander et al., 2019). They are also second messengers that can modulate gene expression and initiate the synthesis of gut peptides and hormones. One of the major SCFAs is propionate, which is three carbons in length (Alexander et al., 2019). It is estimated that in a human being who weighs 85 kg, the gut microbiota produce approximately 29.5 mg/kg of propionate each day *via* fermentation (Morrison and Preston, 2016).

In addition to fermentation, two other sources of propionate are food and the oral microbiome. In 1984, the Food and Drug Administration (FDA) labeled propionate as generally recognized as safe (GRAS) and approved its use for food preservation (U.S. Department of Agriculture, 2008). It is found in a concentration of 0.1 to 0.4% in various foods, including baked goods, dairy products, meat products, puddings, gelatins, and jams (Mani-López et al., 2012; Reis et al., 2012; Tirosh et al., 2019; U.S. Department of Agriculture Technical Advisory Committee, 2002). Therefore, most persons are exposed to dietary sources of propionate every day. It is estimated that in a single meal consisting of processed food, propionate is 0.3% (w/w; Tirosh et al., 2019). Dietary choices thus could potentially impact the amount of propionate in the peripheral circulation. Indeed, Chambers et al. (2017) found an increase in the levels of propionate in the peripheral circulation of healthy adults who were given oral supplements of propionate after an overnight fast. Similarly, Tirosh et al. (2019) found that a meal containing a low dose of propionate led to a significant increase in postprandial plasma levels of propionate in healthy humans. As for the oral microbiome, oral microbiota can produce propionate (Takahashi, 2015). Increased propionate levels are associated with gingivitis and periodontal disease.

When propionate is ingested or generated in the intestine, it makes its way to the liver through the hepatic portal vein (Hoyles et al., 2018). Approximately 90–95% of propionate is used by the liver. The remaining propionate enters the peripheral circulation. Tian et al. (2020) found that the average serum levels of propionate were 2.843 mmol/L (2,843 μM) directly following a 12 h fast in healthy adults between the ages of 20 and 40. In an interesting study, Wolever et al. (1997) found that the average propionate serum levels over 12 h were 3.8 μmol/L (3.8 μM) in young adults and 4.6 μmol/L (4.6 μM) in middle-aged adults. Propionate can cross the blood-brain barrier (BBB). Hoyles et al. (2018) found that there is a propionate free fatty acid receptor 3 (FFAR3) on the endothelium of the human brain. According to the Human Metabolome Database, typical values of propionate in the cerebrospinal fluid (CSF) are 2.8–3.2 μM in adults (Wishart et al., 2017). Propionate is also found in the saliva. According to the Human Metabolome Database, different studies have found different ranges of resting propionate saliva levels, with levels ranging from 1 to 1,089.82 μM (Wishart et al., 2017). Differences in the levels of propionate have been attributed to oral health, gender, and smoking status (Takeda et al., 2009).

Propionate serves several functions in the human body. For instance, propionate promotes enteric smooth muscle contractions and stimulates host defense peptide expression (Mitsui et al., 2005; Sunkara et al., 2012; De Vadder et al., 2014). Deficient levels of propionate have been associated with increased risk for asthma and allergies, highlighting the positive role of propionate in the immune system (Böttcher et al., 2000; Roduit et al., 2018; Ivashkin et al., 2019). Additionally, the metabolism of propionate is associated with glucose production and energy metabolism (Ringer, 1912; Tirosh et al., 2019). Through a series of reactions, propionate is first converted to propionyl-CoA before ultimately being converted to succinyl-CoA (Berg et al., 2002). Succinyl-CoA is a substrate in the TCA cycle. Thus, dietary propionate could perhaps impact the TCA cycle. Perry et al. (2016) found that ingestion of a bolus of propionate increased the concentrations of propionyl-CoA by 100-fold in rodents. Additionally, propionate can participate in the gut-brain axis (Chambers et al., 2015). There is evidence that propionate can affect satiety by stimulating the release of peptide YY (PYY) and glucagon-like peptide-1 (GLP-1). PYY and GLP-1 function to provide a short-term signal of satiety to the brain.

Excess levels of propionate appear to be problematic. One example of the effects of excess propionate is propionic acidemia (PA). This metabolic disorder has been associated with motor impairments, brain atrophy, cognitive impairments, and dementia (Sethi et al., 1989; Morland et al., 2018; Schwoerer et al., 2018). Furthermore, persons with periodontal disease have increased levels of propionate in their saliva and appear to be at an increased risk for developing Alzheimer’s disease (AD; Aimetti et al., 2011; Kamer et al., 2015; Chen et al., 2017). In line with those findings, there is emerging evidence that suggests that excess propionate may play a role in dementia, particularly in AD. Dementia is an age-related disease that is associated with cognitive decline. AD is the most common type of dementia and is characterized by neurofibrillary tangles and β-amyloid plaques (Delacourte, 1994; Hardy and Duff, 1994). According to the Alzheimer’s Association, an estimated 5.8 million individuals currently have AD in the United States (Alzheimer’s Association, 2020). This number is projected to increase to 13.8 million by the year 2050 (Alzheimer’s Association, 2020). In this review article, we will explore the literature that supports the potential role of excess propionate in AD.

## Propionate, Valproate, and Alzheimer’s Disease

Several recent studies offer evidence for a link between propionate and AD. For instance, Figueira et al. (2016) analyzed saliva samples of persons with dementia and healthy controls. They found a 1.35-fold increase in propionate levels in persons with dementia when compared to healthy controls. Yilmaz et al. (2017) analyzed saliva samples from persons with mild cognitive impairment, persons with AD, and healthy controls. They found that the levels of propionate were significantly increased in persons with AD in comparison to healthy controls. Both studies were limited in that they only evaluated saliva samples; however, there is evidence for the validity of using saliva samples. Martin et al. (2018) found that in comparison to plasma levels, saliva levels of oxytocin better correlated with CSF levels. Similarly, Adamashvili et al. (2005) found that saliva levels of human major histocompatibility antigens (HLA) correlated with CSF HLA levels in persons with Multiple Sclerosis. Kennedy et al. (2001) found that epinephrine in saliva appears to originate from both the salivary sympathetic nerves and peripheral circulation. Moreover, Valstar et al. (2020) recently discovered the existence of what they deemed the tubarial glands, which are salivary glands located within the nasopharynx. Several rodent studies link fecal and circulating levels of propionate to AD. Fujii et al. (2019) found that mice given a fecal microbiota transplant from patients with AD had significantly higher levels of propionate in comparison to controls. There is evidence that fecal levels of propionate are positively correlated with circulating levels of propionate in humans (Müller et al., 2019). Additionally, González-Domínguez et al. (2015) found a 1.23-fold increase in propionate in the hippocampus of AD transgenic mice. Similarly, Syeda et al. (2018) found a significantly higher concentration of propionate in the prefrontal cortex of AD transgenic mice in comparison to wild type mice. They also found that AD transgenic mice had significantly higher fecal concentrations of propionate at 6 months of age in comparison to wild type mice.

**Figure 1:**
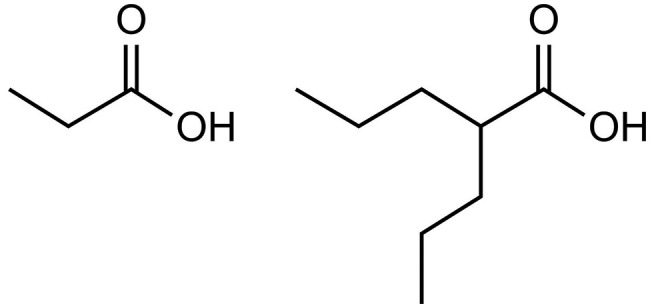
Propionic acid (left) and valproic acid (right).

Valproate provides further evidence for the role of excess propionate in AD. As illustrated by Figure 1, valproate is structurally similar to propionate. Valproate (VPA) and its conjugate acid (valproic acid) are FDA approved medications (under trade names Divalproex, Depakote, Depakote ER, Depakene, Depacon, and Stavzor) for the treatment of epilepsy and bipolar disorder and are prescribed off label for agitation for persons with dementia (Chiu et al., 2013; Baillon et al., 2018). However, a growing body of evidence suggests that VPA causes neurotoxicity that is associated with its metabolism into propionic acid (Farooq et al., 2017). In excessive amounts, propionic acid inhibits a urea cycle enzyme, carbamoyl phosphate synthase, which impairs the body’s ability to excrete ammonia and thus results in hyperammonemia. Once considered only a rare side effect, VPA induced hyperammonemic encephalopathy is now reported frequently in the literature. It produces clinical signs and symptoms that appear to mirror those found in dementia (see Table 1, Kowalski et al., 2013). In a study investigating VPA’s effects on fatty acid metabolism and the urea cycle in schizophrenic patients, Ando et al. (2017) found that 30% developed hyperammonemia (defined as ammonia greater than 47 μmol/L). In a follow-up study by Baddour et al. (2018) on 347 patients treated at a community teaching hospital, the reported incidence of hyperammonemia was found to be 36%, with 43.2% of those patients presenting with clinical symptoms.

**Table 1:** Symptoms of valproate-induced hyperammonemia.

Changes in
1. Orientation
2. Memory
3. Motor function
4. Fatigue
5. Onset or worsening of psychosis
6. Delirium
7. Mood

As further evidence of the neurotoxicity associated with VPA, Armon et al. (1996) found that patients with epilepsy who had been on VPA therapy for at least 1-year experienced reversible cognitive impairments and brain atrophy. Tsai et al. (2016) found that valproic acid treatment increased the risk of developing dementia by 73–95% in patients with bipolar disorder in comparison to patients who were not on valproic acid treatment. Pardoe et al. (2013) compared the brain volume, white matter volume, and parietal lobe thickness of persons with epilepsy who were taking sodium valproate to persons with epilepsy not on sodium valproate treatment and to healthy controls. They found that sodium valproate treatment resulted in brain atrophy, white matter volume loss, and a reduction in parietal lobe thickness. Tariot et al. (2011) explored the effects of VPA on patients with AD. They found that valproate treatment caused greater brain volume loss in comparison to the placebo. In a second study with the same AD patients from the Tariot et al.’s (2011) study, Fleisher et al. (2011) found that VPA caused increased brain volume loss and ventricular expansion in comparison to controls. Furthermore, through the first year of the study, the Mini-Mental State Examination scores implied that VPA treatment led to an accelerated decline in cognition. Taken together, these studies provide further evidence for the role of propionate in AD.

## The Commensal Microbiota, Propionate, and Alzheimer’s Disease

The commensal microbiota is also relevant to the discussion on propionate and AD. Aguirre et al. (2016) and Salonen et al. (2014) found that one type of bacteria called *Bacteroidetes* serves as the principal producers of propionate in the human gut. Specifically, *Bacteroidetes* convert hexose sugars into propionate *via* a succinate pathway (Reichardt et al., 2014). Table 2 illustrates the class, order, family, and genus relevant to the discussion on propionate. There appear to be age-related changes in the human microbiome, particularly changes in the relative abundance of *Bacteroidetes*. For instance, Claesson et al. (2011) found that elderly participants had a greater proportion of *Bacteroides* species than younger adults. Likewise, Odamaki et al. (2016) found that the elderly participants had a significantly higher relative abundance of *Bacteroidetes*, in comparison to infant and adult participants.

**Table 2:** Bacteroidetes.

Phylum	*Bacteroidetes*
Class	*Bacteroidia*
Order	*Bacteroidales*
Family	*Bacteroidaceae*
Genus	*Bacteroides*

*Bacteroidetes* appear to play a role in AD and appear to potentially account for the excess levels of propionate in AD. For instance, Vogt et al. (2017) found that the *Bacteroidetes* phylum was increased in persons with AD when compared to healthy controls. They also found that the levels of several AD markers in CSF were significantly correlated with the relative abundance of the *Bacteroides* genus. Haran et al. (2019) found the *Bacteroides* genus to be increased in persons with AD. Although Liu et al. (2019) did not find the *Bacteroidetes* phylum to be significantly increased in persons with AD, they did find that the level of *Bacteroidetes* was significantly increased in persons at the predementia stage of AD. Wang et al. (2016) found that the *Bacteroidales* order was negatively correlated with spatial learning and memory ability, active avoidance response, and object recognition memory capability in a rodent model of AD. Both Harach et al. (2017) and Kaur et al. (2020) also found that the *Bacteroidetes* phylum was increased in rodent models of AD, while Zhan et al. (2018) found that the *Bacteroidales* order was increased in a rodent model of AD. Harach et al. (2017) found that germ-free generated APPPS1 mice displayed decreased levels of cerebral Aβ42 in comparison to conventionally raised APPPS1 mice. This finding supports a possible role of the gut microbiome in amyloid precursor protein (APP) expression.

In addition to *Bacteriales*, *Actinobacteria* may also play a role in AD. Zhuang et al. (2018) found that the relative abundance of this phylum was increased in the gut of persons with AD, in comparison to healthy controls. *Propionibacterium acnes* (*P. acnes*), which is part of the *Actinobacteria* phylum, is named after its ability to produce propionic acid (Kirschbaum and Kligman, 1963). It is part of the skin, oral, and gut microbiome. It can also cross the BBB (Lu et al., 2019). Concerning AD, *P. acnes* was reportedly found in the cortex of three patients with AD (Kornhuber, 1996). Also, Emery et al. (2017) employing 16S rRNA sequencing analysis to investigate possible bacterial infections in AD brains, consistently found high levels of *P. acnes* in AD samples compared to normal brains under methodological conditions that would make contamination an unlikely explanation for their findings.

## Potential Mechanisms

There is evidence for such a wide array of different mechanisms that excess propionate likely leads to AD by way of a combination of multiple different mechanisms. Probably the most well-studied mechanism of propionate induced neurotoxicity is related to its ability to impair the urea cycle, the principal pathway for nitrogen metabolism. This condition, known as hyperammonemia, occurs in propionic acidemia (PA), an autosomal recessive genetic disease characterized by an abnormal accumulation of propionic acid (Haijes et al., 2019). As aforementioned, hyperammonemia can also occur in patients who are prescribed VPA. In cases of PA with hyperammonemia of ≥360 μmol/L, significant encephalopathy and intellectual disability can occur (Kido et al., 2011). Abnormal accumulation of propionic acid results in excessive propionyl-CoA production, which inhibits N-acetyl-glutamate (NAG) formation (Coude et al., 1979). NAG is important because it activates carbamoyl phosphate synthetase I, which is a key enzyme in the first step of the urea cycle. Propionyl-CoA also inhibits this pathway by depleting hepatic acetyl CoA, which is responsible for NAG synthesis. Propionyl-CoA has a broad impact on metabolism, influencing not only the urea cycle, but also the citric acid cycle and related enzymes, the respiratory chain complex, and the glycine cleavage system. Considering that L-carnitine plays a crucial role in propionic acid metabolism, excessive propionic acid levels inevitably result in L-carnitine deficiency (Maldonado et al., 2016). This further potentiates propionic-acid-mediated neurotoxicity by disrupting β-oxidation pathways and preventing the conversion of propionyl-CoA into the nontoxic and beneficial propionyl carnitine (Roe et al., 1984).

Although acute hyperammonemia can cause encephalopathy, the clinical manifestations of chronic, slightly elevated blood ammonia levels have received relatively little research interest within the field of dementia research (Jin et al., 2018). However, considering the well-known neurotoxic nature of ammonia, it is reasonable to speculate that chronically elevated levels of ammonia might be associated with the development of AD. Indeed, some small clinical studies have reported an association between AD and elevated blood ammonia levels (Fisman et al., 1985, 1989; Branconnier et al., 1986). While ammonia is a normal end product of human tissue metabolism, it is a highly neurotoxic compound at even sub-millimolar concentrations (Marcaida et al., 1992; Roquilly et al., 2013). Thus, ammonia detoxification in organisms is indispensable. In the brain, under normal or hyperammonemic conditions, ATP-dependent formation of glutamine by glutamine synthetase [L-glutamate:ammonia ligase (ADP-forming; E.C.6.3.1.2); GS] is predominantly used for ammonia removal (Norenberg and Martinez-Hernandez, 1979; Cooper and Plum, 1987). In hyperammonemia, astroglia located in proximity to blood-vessels in glutamatergic areas show increased GS protein content in their perivascular processes. Since ammonia freely crosses the BBB and astrocytes are responsible for maintaining the BBB, the presence of GS in the perivascular processes can produce a rapid glutamine synthesis and subsequent release into the blood to limit excess ammonia from circulation. The changes in the distribution of this critical enzyme suggest that the glutamate-glutamine cycle may be differentially impaired in hyperammonemic states (Robinson, 2000; Suárez et al., 2002). Combining a genomic and transcriptomic approach, Bensemain et al. (2009) characterized the induction of the urea cycle metabolic pathway in the brains of AD cases. They found that the expression of the ornithine transcarbamylase (OTC) protein, another key enzyme of the urea cycle, in endothelial cells of AD brain vessels was increased 880% in the CSF of probable AD cases compared with controls. Future studies investigating the relationship between chronically low-grade hyperammonemia and AD should also concurrently measure propionic acid levels in saliva and blood to determine if there is a causal relationship between excess propionic acid levels and hyperammonaemia, as seen in PA and patients treated with VPA.

Another potential mechanism may involve insulin. Studies have shown that SCFAs, especially butyrate, may improve insulin sensitivity (Henagan et al., 2015). However, there is evidence that propionate is not beneficial for insulin sensitivity. Tirosh et al. (2019) investigated the role of propionate in glucose production in humans and in rodents. This study, unlike most other studies on propionate and insulin sensitivity, included participants that were healthy and lean. Other studies have found that propionate can improve insulin sensitivity; however, they are limited in that they either: (1) included unrepresentative delivery or quantities of propionate; and/or (2) included participants who were only overweight or prediabetic (Pingitore et al., 2016; Chambers et al., 2019; Müller et al., 2019). In the Tirosh et al.’s (2019) study, the participants consumed a meal containing an amount of calcium propionate representative of that found in a typical meal consisting of processed foods. The rodents were given a similarly representative amount of propionate in their diet. As for the results, the results imply that orally delivered propionate does not have the same positive effects on insulin sensitivity that are associated with the SCFAs derived from the gut microbiota. In fact, the results imply that orally delivered propionate may instead lead to insulin resistance and glucose intolerance. In the human participants, the propionate-enriched meal leads to increased postprandial levels of insulin. In the rodents, they studied the long term results of orally delivered propionate. The results also imply a role of propionate in insulin resistance. However, it is worth noting that the study is limited in that it only included 14 middle-aged men. Thus, a larger study would be necessary to confirm their results and to elucidate the long-term effects of orally delivered propionate. Darzi et al. (2012) studied the effects of the consumption of bread containing propionate in lean, healthy women, and men. Consistent with the Tirosh et al.’s (2019) study, they found that propionate caused increased postprandial levels of insulin. Moreover, Sanna et al. (2019) found increased fecal levels of propionate to be associated with an increased risk for Type 2 diabetes mellitus. These findings are notable as there is evidence that persons with Type 2 diabetes are at an increased risk for developing AD (Cheng et al., 2011; Madmoli et al., 2019). Additionally, Ciudin et al. (2017) found that Type 2 diabetes was an independent risk factor for the progression of MCI to dementia. Thus, taken together, these studies suggest that insulin resistance may be one mechanism by which excess propionate leads to AD. However, further studies are necessary to clarify this potential mechanism.

As for other mechanisms, propionate has been found to have several other neurotoxic effects, including mitochondrial dysfunction, neuroinflammation, glutamate excitotoxicity, DNA damage, inhibition of Na^+^/K^+^-ATPase, apoptosis of neuronal cells, an increase in oxidative stress, and a decrease in superoxide dismutase activity and both glutathione and serotonin levels (Wyse et al., 1998; Rigo et al., 2006; MacFabe et al., 2008; El-Ansary et al., 2013, 2017; Khalil et al., 2015; Al-Orf et al., 2018). These neurotoxic effects have been associated with AD. For instance, Mandal et al. (2015) found that the antioxidant glutathione was significantly decreased in the brains of persons with MCI and AD. Glutathione levels were also inversely correlated with the severity of the cognitive impairments in the participants. However, future studies are necessary to further clarify the mechanisms by which excess propionate leads to AD.

## Potential Interventions

It is well established that L-carnitine (CAR) supplementation as an adjuvant therapy contributes to the amelioration of blood markers of oxidative damage in patients affected by disorders of excess propionate levels, as well as in the treatment of VPA-induced hyperammonemia (Roe et al., 1984; Ribas et al., 2010; Maldonado et al., 2016; Cutshall et al., 2017). As Maldonado et al. (2016) hypothesized from their study, “*In patients treated with VPA, CAR depletion followed by [acetyl-l-carnitine] ALCAR decrease could be responsible for the increase in the ammonia levels. On the other hand, in the elderly population, serum CAR could be increased due to impaired access to tissues which in turn could result in an ALCAR decrease. This last fact could lead to ammonia impaired elimination. Perhaps higher ammonia levels and ALCAR deficit could be responsible for the cognitive and neurodegenerative diseases found in the elderly.”* Indeed, numerous clinical trial studies have investigated the cognitive therapeutic benefits of L-carnitine and acetyl-l-carnitine treatment in AD (Spagnoli et al., 1991; Montgomery et al., 2003). However, only recently have investigators considered explaining their putative therapeutic benefits in the context of reducing hyperammonemia in neurological disorders of the elderly (Maldonado et al., 2016, 2020). And, to our knowledge, no one until now has considered excess propionate production *via* a bacterial infection as a possible causal process resulting in prolonged low-grade hyperammonemia.

The metabolic pathways associated with the breakdown of propionate may also offer points for intervention. For example, vitamin B-12 is a cofactor in the conversion of propionate to succinyl-CoA (Berg et al., 2002). Revtovich et al. (2019) studied vitamin B-12 and propionate levels in *Caenorhabditis elegans*. Their results support vitamin B-12 playing a role in the breakdown of propionate. They also explored vitamin B-12 supplementation concerning mitochondrial health, since excess propionate leads to mitochondrial dysfunction. They found that vitamin B-12 supplementation in *C. elegans* resulted in improved mitochondrial health. Concerning AD, decreased vitamin B-12 levels appear to be linked to AD (Ma et al., 2017). Thus, perhaps decreased vitamin B-12 could be another potential cause of the excess propionate. Douaud et al. (2013) found that vitamin B-12 supplementation was able to reduce cerebral atrophy in the gray matter regions that are affected by AD. This reduction was significant, as it was a 7-fold reduction. In addition to vitamin B-12, propionyl-CoA carboxylase may also be a viable target for intervention. Like vitamin B-12, this enzyme is also involved in the conversion of propionate to succinyl-coenzyme A (Berg et al., 2002). Dysregulated propionyl-CoA carboxylase can lead to increased levels of propionate (Morland et al., 2018). Cuadrado-Tejedor et al. (2013) found that older transgenic AD mice displayed deregulated propionyl-CoA carboxylase. Therefore, propionyl-CoA carboxylase, along with vitamin B-12, warrants future study.

Another potential intervention may be the antioxidant TEMPOL(4-hydroxy-2,2,6,6-tetramethylpiperidine-N-oxyl), which is a small (MW 172 Da) stable nitroxide radical that can readily permeate biological membranes. Cai et al. (2016) found that TEMPOL could reduce cecal and fecal concentrations of propionate in obese rodents. In relation to AD, Ali et al. (2016) and Khallaf et al. (2017) found that TEMPOL was able to reduce neuroinflammation, cognitive impairments, and amyloidogenesis in the rodents. Ali et al. (2016) also found that TEMPOL was able to reduce oxido-nitrosative stress, which has been found to play a role in ammonia toxicity (Skowrońska and Albrecht, 2013). This is of particular interest as propionate can impair the urea cycle and lead to hyperammonemia. Thus, this antioxidant warrants further study, especially concerning its effects on propionate and its effects on oxido-nitrosative stress.

Butyrate, an SCFA, may be another potential intervention. Butyrate appears to have neuroprotective effects and has been indicated as a viable treatment for neurological disorders, such as Parkinson’s disease (Liu et al., 2017). Concerning AD, Govindarajan et al. (2011) found that butyrate improved cognition in a rodent model of AD. Cleophas et al. (2019) found that butyrate supplementation reduced fecal levels of propionate in both lean and obese men. Thus, like vitamin B-12 and potentially TEMPOL, butyrate supplementation may be a viable method to reduce propionate levels. Yet, further research is needed to clarify this potential role of butyrate. Further work would especially need to study the effect of butyrate on circulating levels of propionate.

Diet may be a target for intervention, as diet could directly impact one’s intake of propionate. Furthermore, diet can impact the levels of the *Bacteroidetes* phylum. In comparison to non-Western diets, a Western diet, which consists of high protein and fat, was found to increase the levels of *Bacteroidetes* or *Bacteroides* in several studies (Filippo et al., 2010; Wu et al., 2011; David et al., 2013; Yeagle, 2015; Heinritz et al., 2016). Furthermore, keeping in mind that propionate is used as a food preservative, a diet that features low consumption of foods with propionate may be another viable intervention. Some promising dietary interventions on autism spectrum disorders (ASD) show some evidence for dietary interventions for propionate. ASD appears to be another neurological disease associated with excess propionate. Multiple studies have found that propionate causes ASD-like behaviors in rodents (MacFabe et al., 2011; Kamen et al., 2018; Mepham et al., 2019; Shams et al., 2019). Additionally, Angelis et al. (2013) and Wang et al. (2012) found that children with ASD had higher levels of propionate in comparison to healthy controls. As for dietary interventions, in several studies, participants with ASD experienced improvements in their symptoms when placed on a dairy-free and gluten-free diet (Knivsberg et al., 2002; Whiteley et al., 2010; Ghalichi et al., 2016; El-Rashidy et al., 2017). Additionally, as a preventative measure, replacing propionate with other compounds for food preservation may be beneficial.

## Conclusion

Propionate serves important functions in the body. However, excess levels of propionate may play a role in AD. The cause of the excessive levels of propionate could be related to diet, medication use, the commensal microbiota, or potentially related to propionate metabolism. Future studies should aim to clarify the cause of the excess levels. There are multiple mechanisms by which propionate may lead to AD, including glutamate excitotoxicity and hyperammonemia. The mechanisms offer potential points for intervention.

## Author Contributions

JK reviewed the literature and wrote this article. DS and RS edited this article. All authors contributed to the article and approved the submitted version.

## Conflict of Interest

The authors declare that the research was conducted in the absence of any commercial or financial relationships that could be construed as a potential conflict of interest.
